# 4-Chloro-3-ethyl­phenol

**DOI:** 10.1107/S1600536814013919

**Published:** 2014-06-21

**Authors:** Sean H. Majer, Joseph M. Tanski

**Affiliations:** a Department of Chemistry, Vassar College, Poughkeepsie, NY 12604, USA

**Keywords:** crystal structure

## Abstract

The title compound, C_8_H_9_ClO, packs with two independent mol­ecules in the asymmetric unit, without significant differences in corresponding bond lengths and angles, with the ethyl group in each oriented nearly perpendicular to the aromatic ring having ring-to-side chain torsion angles of 81.14 (18) and −81.06 (19)°. In the crystal, mol­ecules form an O—H⋯O hydrogen-bonded chain extending along the *b*-axis direction, through the phenol groups in which the H atoms are disordered. These chains pack together in the solid state, giving a sheet lying parallel to (001), *via* an offset face-to-face π-stacking inter­action characterized by a centroid–centroid distance of 3.580 (1) Å, together with a short inter­molecular Cl⋯Cl contact [3.412 (1) Å].

## Related literature   

For information regarding the synthesis of 4-chloro-3-ethyl­phenol, see the following patents: Awano *et al.* (1987 ▶) or Schroetter *et al.* (1977 ▶). For applications in biological systems, see: Gerbershagen *et al.* (2005 ▶); Low *et al.* (1997 ▶). For similar chlorinated phenols, see: Cox (1995 ▶, 2003 ▶); Oswald *et al.* (2005 ▶). For more information on π-stacking, see: Lueckheide *et al.* (2013 ▶) and on halogen–halogen inter­actions, see: Pedireddi *et al.* (1994 ▶).
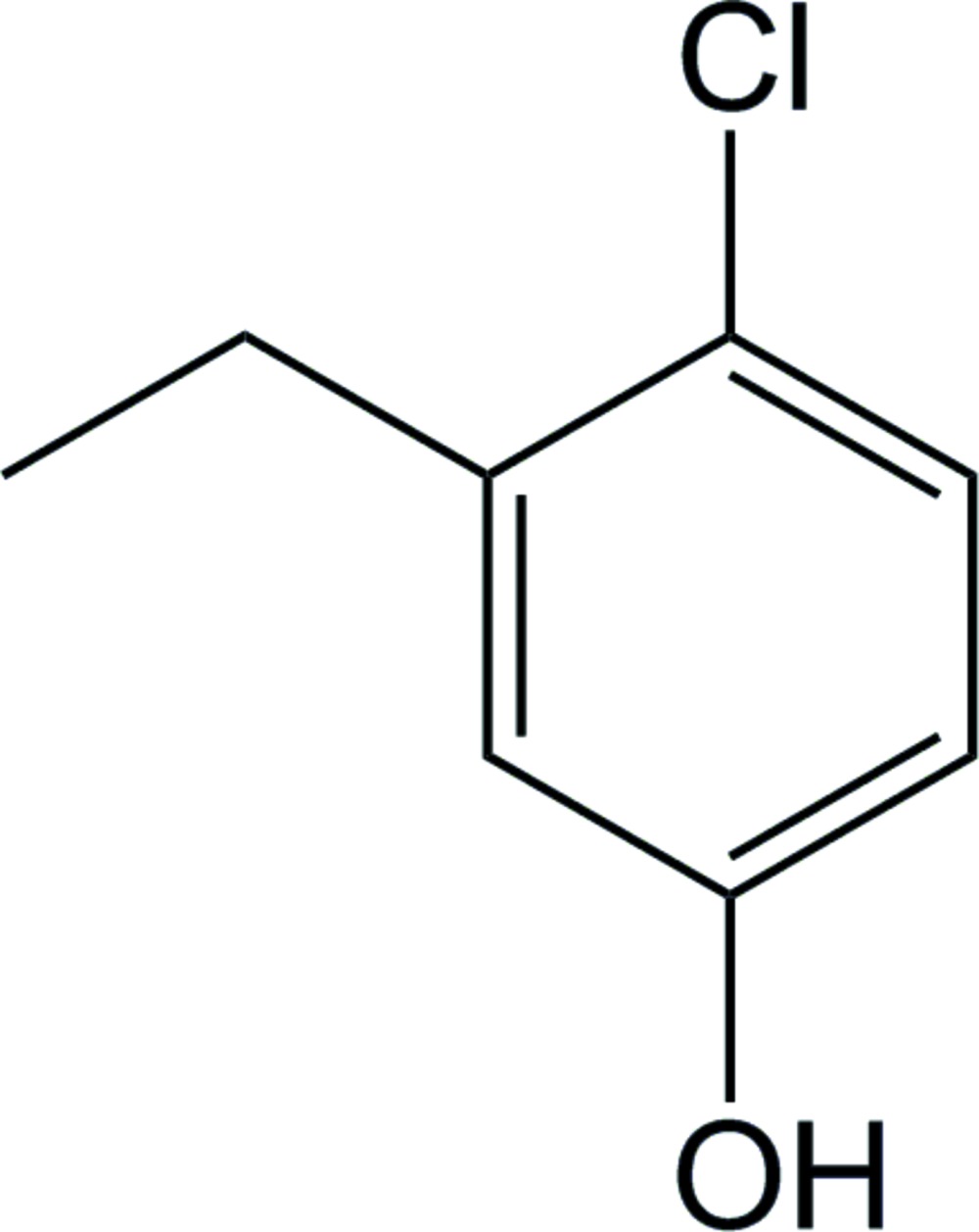


## Experimental   

### 

#### Crystal data  

C_8_H_9_ClO
*M* *_r_* = 156.60
Triclinic, 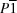

*a* = 7.5580 (7) Å
*b* = 8.6854 (8) Å
*c* = 12.2520 (11) Å
α = 78.363 (1)°
β = 78.762 (1)°
γ = 80.355 (1)°
*V* = 765.72 (12) Å^3^
*Z* = 4
Mo *K*α radiation
μ = 0.42 mm^−1^
*T* = 125 K
0.20 × 0.15 × 0.10 mm

#### Data collection  

Bruker APEXII CCD diffractometer
Absorption correction: multi-scan (*SADABS*; Bruker, 2007 ▶) *T*_min_ = 0.910, *T*_max_ = 0.949
17904 measured reflections
4656 independent reflections
4176 reflections with *I* > 2σ(*I*)
*R*_int_ = 0.019

#### Refinement  

*R*[*F* ^2^ > 2σ(*F* ^2^)] = 0.037
*wR*(*F* ^2^) = 0.107
*S* = 1.13
4656 reflections
183 parameters
4 restraints
H-atom parameters constrained
Δρ_max_ = 0.48 e Å^−3^
Δρ_min_ = −0.26 e Å^−3^

### 

Data collection: *APEX2* (Bruker, 2007 ▶); cell refinement: *SAINT* (Bruker, 2007 ▶); data reduction: *SAINT*; program(s) used to solve structure: *SHELXS97* (Sheldrick, 2008 ▶); program(s) used to refine structure: *SHELXL97* (Sheldrick, 2008 ▶); molecular graphics: *SHELXTL* (Sheldrick, 2008 ▶); software used to prepare material for publication: *SHELXTL*, *OLEX2* (Dolomanov *et al.*, 2009 ▶) and *Mercury* (Macrae *et al.*, 2006 ▶).

## Supplementary Material

Crystal structure: contains datablock(s) I, New_Global_Publ_Block. DOI: 10.1107/S1600536814013919/zs2303sup1.cif

Structure factors: contains datablock(s) I. DOI: 10.1107/S1600536814013919/zs2303Isup2.hkl

Supporting information file. DOI: 10.1107/S1600536814013919/zs2303Isup3.cml

CCDC reference: 1008296

Additional supporting information:  crystallographic information; 3D view; checkCIF report

## Figures and Tables

**Table 1:** Hydrogen-bond geometry (Å, °)

*D*—H⋯*A*	*D*—H	H⋯*A*	*D*⋯*A*	*D*—H⋯*A*
O1—H1⋯O1^i^	0.81	1.97	2.708 (3)	152
O1—H1*A*⋯O2^i^	0.81	1.86	2.6642 (17)	171
O2—H2⋯O1^i^	0.81	1.86	2.6642 (17)	168
O2—H2*A*⋯O2^ii^	0.82	1.91	2.704 (2)	166

Symmetry codes: (i) 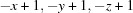; (ii) 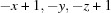.

**Fig. 1.**
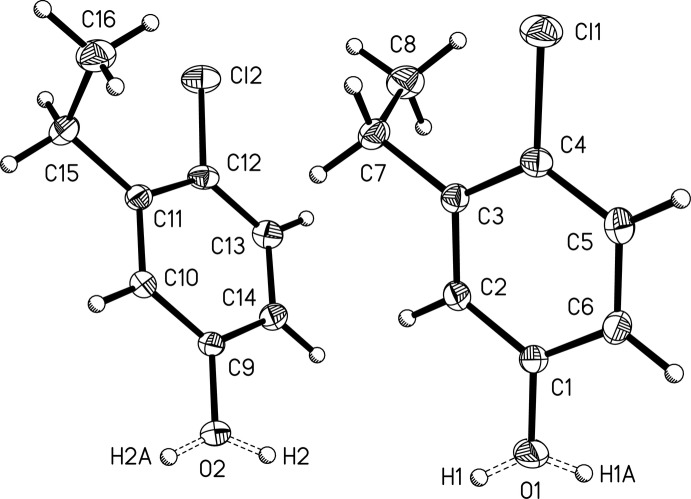
A view of the two independent molecules of the title compound with the atom numbering scheme. Displacement ellipsoids are shown at the 50% probability level. The disordered phenolic hydrogen atoms are represented with dashed open bonds.

**Fig. 2.**
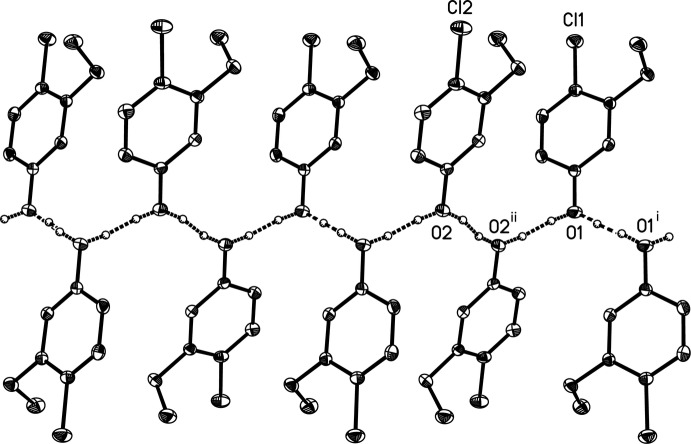
A view of the one-dimensional hydrogen-bonded chain extending along b, with displacement ellipsoids shown at the 50% probability level. For symmetry codes (i) and (ii), see Table 1.

**Fig. 3.**
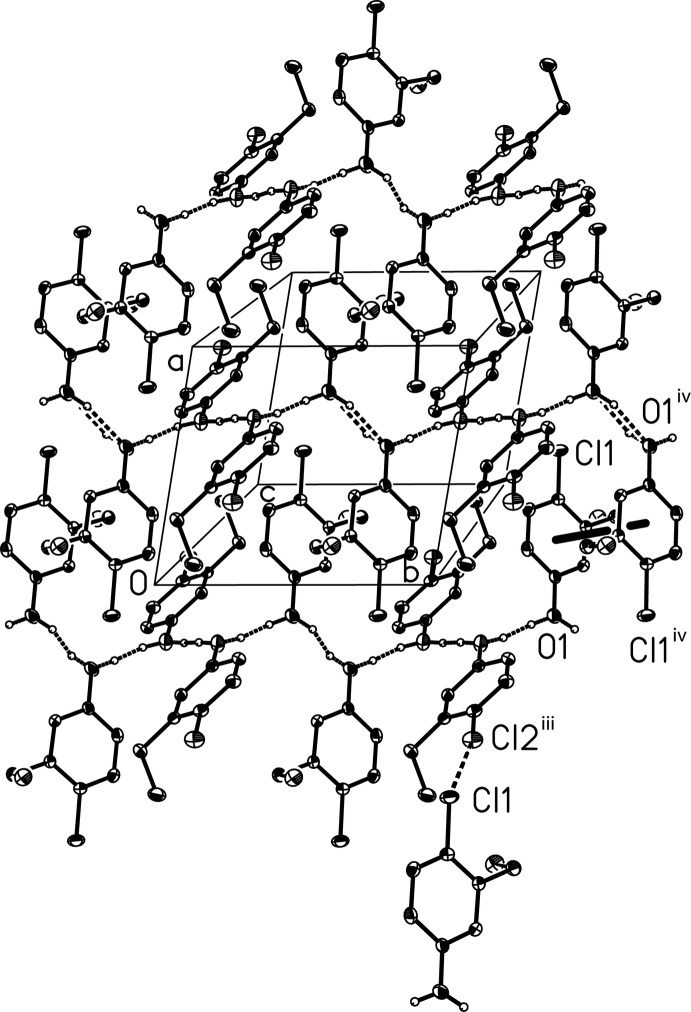
A view of the offset face-to-face π-stacking in the structure of title compound, with a solid line indicating one interaction and a dashed line indicating one of the Cl1···Cl2 interactions. For symmetry codes: (iii) -x, -y + 1, -z; (iv): -x, -y + 1, -z + 1. Displacement ellipsoids are shown at the 50% probability level.

## supplementary crystallographic information

### S1. Comment

4-Chloro-3-ethylphenol, the title compound, can be synthesized by chlorination of 3-ethylphenol by SO_2_Cl_2_ in the presence of FeCl_3_ in CCl_4_ (Awano *et al.*, 1987) or by adding the hydroxyl group to 1-ethyl-2-nitrobenzene followed by an acidic workup and a Sandmeyer reaction with CuCl (Schroetter *et al.*, 1977*)*. The title compound has been found to be useful in multiple biological applications, including testing the contracture in malignant hypothermia skeletal tissue (Low *et al.*, 1997) and in biological activity on Ca^2+^ deposits in muscle cells (Gerbershagen *et al.*, 2005).

The two independent molecules of the title compound in the asymmetric unit (Fig. 1) exhibit C—Cl bond lengths of 1.7430 (15) and 1.7469 (15) Å, and C—O bond lengths of 1.3751 (18) and 1.3778 (17) Å, respectively. These are in very close agreement with analogous bond lengths in the stuctures of 4-chlorophenol (Oswald *et al.*, 2005), 4-chloro-3-methylphenol (Cox, 2003), and 4-chloro-3,5-dimethylphenol (Cox, 1995). The ethyl group is rotated nearly perpendicular to the plane of the ring for each independent molecule, displaying very similar torsion angles of 81.14 (18)° (C4—C3—C7—C8) and -81.06 (19)° (C12—C11—C15—C16). The structure forms a one-dimensional O—H···O hydrogen-bonded chain through the phenol groups, in which the phenol protons are 50% rotationally disordered (Fig. 2). These chains run parallel to the crystallographic *b*-axis. Each independent molecule forms hydrogen bonds with a neighboring equivalent independent molecule, with an oxygen–oxygen distance (O1···O1^i^) of 2.708 (3) Å and an oxygen–oxygen distance (O2···O2^ii^) of 2.704 (2) Å [for symmetry codes (i) and (ii), see Table 1]. These pairwise dimers are hydrogen-bonded to one another resulting in a third unique hydrogen bond, (O1···O2^i^), with length 2.6642 (17) Å. A similar hydrogen-bonding motif is found in the ordered one-dimensional hydrogen bonding chain in the structure of 4-chloro-3-methylphenol (Cox, 2003), where the O···O distances are similar at 2.711 (2) and 2.714 (2) Å. Unlike 4-chloro-3-methylphenol, where the planes of the aromatic units on each side of the hydrogen-bonded chain are parallel, in the the title compound they form a herringbone (edge-to-face or T) motif.

Neighboring hydrogen-bonded chains pack together in the solid state to form a two-dimensional sheet parallel to the 0 0 1 plane *via* an offset face-to-face π-stacking interaction of one of the two independent molecules, whereas the other molecule does not engage in π-stacking (Fig. 3). The π-stacking is characterized by a centroid-to-centroid distance of 3.580 (1) Å, a plane-to-centroid distance of 3.410 (1) Å, and a ring offset or ring-slipage distance of 1.092 (3) Å (Lueckheide *et al.*, 2013). Neighboring sheets are further linked by a short intermolecular chlorine–chlorine contact (Cl1···Cl2^iii^) of 3.412 (1) Å, which is less than the sum of the van der Waals radii of 3.50 Å for chlorine–chlorine interactions (Pedireddi *et al.*, 1994). For symmetry code (iii): -*x*, -*y* + 1, -*z*.

### S2. Experimental

4-Chloro-3-ethylphenol was purchased from Aldrich Chemical Company, USA, and recrystallized from hexanes.

### S3. Refinement

All non-hydrogen atoms were refined anisotropically. Hydrogen atoms on carbon were included in calculated positions and refined using a riding model with C–H = 0.95, 0.98 and 0.99 Å and *U*_iso_(H) = 1.2, 1.5 and 1.2 × *U*_eq_(C) of the aryl, methyl and methylene C-atoms, respectively. The positions of the disordered phenolic hydrogen atoms were found in the difference map and refined semi-freely at 50% occupancy using a distance restraint d(O–H) = 0.84 Å, and *U*_iso_(H) = 1.2× *U*_eq_(O).

### Crystal data

**Table:**

C_8_H_9_ClO	*Z* = 4
*M**_r_* = 156.60	*F*(000) = 328
Triclinic, *P*1	*D*_x_ = 1.358 Mg m^−^^3^
Hall symbol: -P 1	Mo *K*α radiation, λ = 0.71073 Å
*a* = 7.5580 (7) Å	Cell parameters from 9958 reflections
*b* = 8.6854 (8) Å	θ = 2.7–30.5°
*c* = 12.2520 (11) Å	µ = 0.42 mm^−^^1^
α = 78.363 (1)°	*T* = 125 K
β = 78.762 (1)°	Block, colourless
γ = 80.355 (1)°	0.20 × 0.15 × 0.10 mm
*V* = 765.72 (12) Å^3^	

### Data collection

**Table:**

Bruker APEXII CCD diffractometer	4656 independent reflections
Radiation source: fine-focus sealed tube	4176 reflections with *I* > 2σ(*I*)
Graphite monochromator	*R*_int_ = 0.019
φ and ω scans	θ_max_ = 30.5°, θ_min_ = 1.7°
Absorption correction: multi-scan (*SADABS*; Bruker, 2007)	*h* = −10→10
*T*_min_ = 0.910, *T*_max_ = 0.949	*k* = −12→12
17904 measured reflections	*l* = −17→17

### Refinement

**Table:**

Refinement on *F*^2^	Primary atom site location: structure-invariant direct methods
Least-squares matrix: full	Secondary atom site location: difference Fourier map
*R*[*F*^2^ > 2σ(*F*^2^)] = 0.037	Hydrogen site location: inferred from neighbouring sites
*wR*(*F*^2^) = 0.107	H-atom parameters constrained
*S* = 1.13	*w* = 1/[σ^2^(*F*_o_^2^) + (0.0336*P*)^2^ + 0.751*P*] where *P* = (*F*_o_^2^ + 2*F*_c_^2^)/3
4656 reflections	(Δ/σ)_max_ < 0.001
183 parameters	Δρ_max_ = 0.48 e Å^−^^3^
4 restraints	Δρ_min_ = −0.26 e Å^−^^3^

### Special details

**Table:**

Geometry. All e.s.d.'s (except the e.s.d. in the dihedral angle between two l.s. planes) are estimated using the full covariance matrix. The cell e.s.d.'s are taken into account individually in the estimation of e.s.d.'s in distances, angles and torsion angles; correlations between e.s.d.'s in cell parameters are only used when they are defined by crystal symmetry. An approximate (isotropic) treatment of cell e.s.d.'s is used for estimating e.s.d.'s involving l.s. planes.
Refinement. Refinement of *F*^2^ against ALL reflections. The weighted *R*-factor *wR* and goodness of fit *S* are based on *F*^2^, conventional *R*-factors *R* are based on *F*, with *F* set to zero for negative *F*^2^. The threshold expression of *F*^2^ > σ(*F*^2^) is used only for calculating *R*-factors(gt) *etc*. and is not relevant to the choice of reflections for refinement. *R*-factors based on *F*^2^ are statistically about twice as large as those based on *F*, and *R*- factors based on ALL data will be even larger.

### Fractional atomic coordinates and isotropic or equivalent isotropic displacement parameters (Å^2^)

**Table:**

	*x*	*y*	*z*	*U*_iso_*/*U*_eq_	Occ. (<1)
Cl1	−0.28100 (5)	0.74811 (5)	0.30473 (4)	0.02708 (10)	
O1	0.36924 (19)	0.62233 (15)	0.52135 (12)	0.0328 (3)	
H1	0.4258	0.5340	0.5280	0.039*	0.50
H1A	0.4024	0.7019	0.5320	0.039*	0.50
C1	0.2194 (2)	0.65320 (17)	0.46838 (13)	0.0186 (3)	
C2	0.20646 (19)	0.56371 (16)	0.38898 (12)	0.0173 (3)	
H2B	0.3035	0.4836	0.3702	0.021*	
C3	0.05376 (19)	0.58914 (16)	0.33626 (11)	0.0157 (2)	
C4	−0.08454 (19)	0.70975 (17)	0.36563 (12)	0.0171 (3)	
C5	−0.0719 (2)	0.80067 (17)	0.44421 (13)	0.0192 (3)	
H5A	−0.1676	0.8822	0.4622	0.023*	
C6	0.0802 (2)	0.77258 (17)	0.49633 (12)	0.0197 (3)	
H6A	0.0894	0.8340	0.5505	0.024*	
C7	0.0464 (2)	0.49276 (18)	0.24819 (13)	0.0207 (3)	
H7A	−0.0793	0.4686	0.2557	0.025*	
H7B	0.1272	0.3910	0.2611	0.025*	
C8	0.1054 (3)	0.5813 (2)	0.12819 (13)	0.0276 (3)	
H8A	0.1021	0.5144	0.0733	0.041*	
H8B	0.2294	0.6062	0.1207	0.041*	
H8C	0.0223	0.6799	0.1140	0.041*	
Cl2	0.36637 (6)	0.24774 (5)	−0.04071 (3)	0.02827 (10)	
O2	0.53688 (18)	0.12745 (14)	0.41968 (10)	0.0271 (3)	
H2	0.5511	0.2054	0.4427	0.033*	0.50
H2A	0.5219	0.0420	0.4598	0.033*	0.50
C9	0.4935 (2)	0.15527 (17)	0.31266 (12)	0.0171 (3)	
C10	0.37918 (19)	0.06378 (16)	0.28560 (12)	0.0172 (3)	
H10A	0.3293	−0.0170	0.3419	0.021*	
C11	0.33641 (19)	0.08887 (17)	0.17674 (12)	0.0172 (3)	
C12	0.4129 (2)	0.20931 (18)	0.09713 (12)	0.0188 (3)	
C13	0.5254 (2)	0.30228 (18)	0.12387 (13)	0.0204 (3)	
H13A	0.5744	0.3839	0.0679	0.024*	
C14	0.5662 (2)	0.27599 (18)	0.23228 (13)	0.0195 (3)	
H14A	0.6426	0.3394	0.2513	0.023*	
C15	0.2080 (2)	−0.00900 (19)	0.15052 (14)	0.0233 (3)	
H15A	0.2089	−0.1105	0.2046	0.028*	
H15B	0.2514	−0.0339	0.0736	0.028*	
C16	0.0132 (2)	0.0767 (2)	0.15729 (16)	0.0292 (3)	
H16A	−0.0649	0.0098	0.1385	0.044*	
H16B	0.0116	0.1770	0.1036	0.044*	
H16C	−0.0320	0.0981	0.2341	0.044*	

### Atomic displacement parameters (Å^2^)

**Table:**

	*U*^11^	*U*^22^	*U*^33^	*U*^12^	*U*^13^	*U*^23^
Cl1	0.01775 (17)	0.0333 (2)	0.0317 (2)	0.00267 (14)	−0.00926 (14)	−0.00914 (16)
O1	0.0381 (7)	0.0216 (5)	0.0469 (8)	−0.0047 (5)	−0.0307 (6)	−0.0013 (5)
C1	0.0229 (7)	0.0145 (6)	0.0204 (6)	−0.0042 (5)	−0.0092 (5)	−0.0007 (5)
C2	0.0173 (6)	0.0147 (6)	0.0199 (6)	−0.0004 (5)	−0.0041 (5)	−0.0032 (5)
C3	0.0172 (6)	0.0154 (6)	0.0147 (6)	−0.0028 (5)	−0.0022 (5)	−0.0027 (5)
C4	0.0152 (6)	0.0188 (6)	0.0172 (6)	−0.0018 (5)	−0.0030 (5)	−0.0029 (5)
C5	0.0201 (6)	0.0165 (6)	0.0199 (6)	−0.0011 (5)	−0.0007 (5)	−0.0042 (5)
C6	0.0262 (7)	0.0162 (6)	0.0180 (6)	−0.0039 (5)	−0.0045 (5)	−0.0044 (5)
C7	0.0224 (7)	0.0223 (7)	0.0195 (6)	−0.0016 (5)	−0.0051 (5)	−0.0084 (5)
C8	0.0329 (8)	0.0333 (8)	0.0170 (7)	0.0006 (7)	−0.0055 (6)	−0.0085 (6)
Cl2	0.0300 (2)	0.0399 (2)	0.01504 (16)	−0.00312 (16)	−0.00605 (13)	−0.00440 (14)
O2	0.0433 (7)	0.0205 (5)	0.0222 (5)	−0.0027 (5)	−0.0190 (5)	−0.0029 (4)
C9	0.0188 (6)	0.0160 (6)	0.0173 (6)	0.0007 (5)	−0.0071 (5)	−0.0035 (5)
C10	0.0184 (6)	0.0152 (6)	0.0180 (6)	−0.0014 (5)	−0.0048 (5)	−0.0022 (5)
C11	0.0161 (6)	0.0174 (6)	0.0194 (6)	0.0012 (5)	−0.0054 (5)	−0.0063 (5)
C12	0.0182 (6)	0.0240 (7)	0.0140 (6)	0.0010 (5)	−0.0042 (5)	−0.0045 (5)
C13	0.0185 (6)	0.0232 (7)	0.0183 (6)	−0.0040 (5)	−0.0016 (5)	−0.0011 (5)
C14	0.0178 (6)	0.0200 (6)	0.0220 (7)	−0.0036 (5)	−0.0052 (5)	−0.0039 (5)
C15	0.0236 (7)	0.0223 (7)	0.0280 (8)	−0.0042 (6)	−0.0098 (6)	−0.0075 (6)
C16	0.0220 (7)	0.0332 (9)	0.0340 (9)	−0.0055 (6)	−0.0099 (6)	−0.0031 (7)

### Geometric parameters (Å, º)

**Table:**

Cl1—C4	1.7430 (15)	Cl2—C12	1.7469 (15)
O1—C1	1.3751 (18)	O2—C9	1.3778 (17)
O1—H1	0.8098	O2—H2	0.8144
O1—H1A	0.8145	O2—H2A	0.8150
C1—C2	1.388 (2)	C9—C10	1.391 (2)
C1—C6	1.391 (2)	C9—C14	1.392 (2)
C2—C3	1.395 (2)	C10—C11	1.399 (2)
C2—H2B	0.9500	C10—H10A	0.9500
C3—C4	1.3993 (19)	C11—C12	1.397 (2)
C3—C7	1.5072 (19)	C11—C15	1.508 (2)
C4—C5	1.388 (2)	C12—C13	1.388 (2)
C5—C6	1.385 (2)	C13—C14	1.388 (2)
C5—H5A	0.9500	C13—H13A	0.9500
C6—H6A	0.9500	C14—H14A	0.9500
C7—C8	1.535 (2)	C15—C16	1.529 (2)
C7—H7A	0.9900	C15—H15A	0.9900
C7—H7B	0.9900	C15—H15B	0.9900
C8—H8A	0.9800	C16—H16A	0.9800
C8—H8B	0.9800	C16—H16B	0.9800
C8—H8C	0.9800	C16—H16C	0.9800
			
C1—O1—H1	119.3	C9—O2—H2	115.6
C1—O1—H1A	113.5	C9—O2—H2A	118.3
H1—O1—H1A	126.1	H2—O2—H2A	124.2
O1—C1—C2	119.94 (14)	O2—C9—C10	120.42 (13)
O1—C1—C6	119.55 (14)	O2—C9—C14	118.89 (13)
C2—C1—C6	120.51 (13)	C10—C9—C14	120.69 (13)
C1—C2—C3	121.26 (13)	C9—C10—C11	121.06 (13)
C1—C2—H2B	119.4	C9—C10—H10A	119.5
C3—C2—H2B	119.4	C11—C10—H10A	119.5
C2—C3—C4	117.27 (13)	C12—C11—C10	117.20 (13)
C2—C3—C7	119.86 (13)	C12—C11—C15	122.80 (13)
C4—C3—C7	122.82 (13)	C10—C11—C15	119.97 (13)
C5—C4—C3	121.79 (13)	C13—C12—C11	122.05 (13)
C5—C4—Cl1	117.97 (11)	C13—C12—Cl2	117.98 (12)
C3—C4—Cl1	120.23 (11)	C11—C12—Cl2	119.97 (11)
C6—C5—C4	120.02 (13)	C14—C13—C12	120.01 (14)
C6—C5—H5A	120.0	C14—C13—H13A	120.0
C4—C5—H5A	120.0	C12—C13—H13A	120.0
C5—C6—C1	119.14 (13)	C13—C14—C9	118.97 (14)
C5—C6—H6A	120.4	C13—C14—H14A	120.5
C1—C6—H6A	120.4	C9—C14—H14A	120.5
C3—C7—C8	111.60 (13)	C11—C15—C16	112.21 (13)
C3—C7—H7A	109.3	C11—C15—H15A	109.2
C8—C7—H7A	109.3	C16—C15—H15A	109.2
C3—C7—H7B	109.3	C11—C15—H15B	109.2
C8—C7—H7B	109.3	C16—C15—H15B	109.2
H7A—C7—H7B	108.0	H15A—C15—H15B	107.9
C7—C8—H8A	109.5	C15—C16—H16A	109.5
C7—C8—H8B	109.5	C15—C16—H16B	109.5
H8A—C8—H8B	109.5	H16A—C16—H16B	109.5
C7—C8—H8C	109.5	C15—C16—H16C	109.5
H8A—C8—H8C	109.5	H16A—C16—H16C	109.5
H8B—C8—H8C	109.5	H16B—C16—H16C	109.5
			
O1—C1—C2—C3	178.12 (13)	O2—C9—C10—C11	−178.90 (13)
C6—C1—C2—C3	−0.8 (2)	C14—C9—C10—C11	1.0 (2)
C1—C2—C3—C4	0.8 (2)	C9—C10—C11—C12	−0.1 (2)
C1—C2—C3—C7	178.38 (13)	C9—C10—C11—C15	−178.34 (13)
C2—C3—C4—C5	−0.2 (2)	C10—C11—C12—C13	−0.6 (2)
C7—C3—C4—C5	−177.72 (14)	C15—C11—C12—C13	177.52 (14)
C2—C3—C4—Cl1	−179.38 (11)	C10—C11—C12—Cl2	179.89 (11)
C7—C3—C4—Cl1	3.11 (19)	C15—C11—C12—Cl2	−2.0 (2)
C3—C4—C5—C6	−0.3 (2)	C11—C12—C13—C14	0.5 (2)
Cl1—C4—C5—C6	178.84 (11)	Cl2—C12—C13—C14	−179.95 (12)
C4—C5—C6—C1	0.3 (2)	C12—C13—C14—C9	0.3 (2)
O1—C1—C6—C5	−178.70 (14)	O2—C9—C14—C13	178.84 (13)
C2—C1—C6—C5	0.2 (2)	C10—C9—C14—C13	−1.1 (2)
C2—C3—C7—C8	−96.30 (16)	C12—C11—C15—C16	−81.06 (19)
C4—C3—C7—C8	81.14 (18)	C10—C11—C15—C16	97.03 (17)

### Hydrogen-bond geometry (Å, º)

**Table:**

*D*—H···*A*	*D*—H	H···*A*	*D*···*A*	*D*—H···*A*
O1—H1···O1^i^	0.81	1.97	2.708 (3)	152
O1—H1*A*···O2^i^	0.81	1.86	2.6642 (17)	171
O2—H2···O1^i^	0.81	1.86	2.6642 (17)	168
O2—H2*A*···O2^ii^	0.82	1.91	2.704 (2)	166

Symmetry codes: (i) −*x*+1, −*y*+1, −*z*+1; (ii) −*x*+1, −*y*, −*z*+1.
